# Fmoc-Diphenylalanine Hydrogels: Optimization of Preparation Methods and Structural Insights

**DOI:** 10.3390/ph15091048

**Published:** 2022-08-25

**Authors:** Carlo Diaferia, Elisabetta Rosa, Giancarlo Morelli, Antonella Accardo

**Affiliations:** Department of Pharmacy and Interuniversity Research Centre on Bioactive Peptides (CIRPeB), University of Naples “Federico II”, Via Montesano 49, 80131 Naples, Italy

**Keywords:** Fmoc-FF, peptide hydrogels, peptide materials, hydrogel preparation, diphenylalanine

## Abstract

Hydrogels (HGs) are tri-dimensional materials with a non-Newtonian flow behaviour formed by networks able to encapsulate high amounts of water or other biological fluids. They can be prepared using both synthetic or natural polymers and their mechanical and functional properties may change according to the preparation method, the solvent, the pH, and to others experimental parameters. Recently, many short and ultra-short peptides have been investigated as building blocks for the formulation of biocompatible hydrogels suitable for different biomedical applications. Due to its simplicity and capability to gel in physiological conditions, Fmoc-FF dipeptide is one of the most studied peptide hydrogelators. Although its identification dates to 15 ago, its behaviour is currently studied because of the observation that the final material obtained is deeply dependent on the preparation method. To collect information about their formulation, here are reported some different strategies adopted until now for the Fmoc-FF HG preparation, noting the changes in the structural arrangement and behaviour in terms of stiffness, matrix porosity, and stability induced by the different formulation strategy on the final material.

## 1. Introduction

Researchers have directed their gaze towards nature since the analysis of biologically relevant structures, elements, and processes can embody a motivating well for inspiration. On the evidence that numerous structures are the consequence of natural self-organization of proteinaceous materials, peptide-based building blocks and their analogues have begun to be studied [1,2,3,4]. In addition, supramolecular aggregates and misfolding protein materials were found as pathological hallmarks of major human illnesses too, including bovine spongiform encephalopathy (mad cow disease), Creutzfeldt–Jakob disease, Alzheimer’s disease, Huntington’s, and Parkinson’s diseases [5]. Although first identified as pathological entities, a new research line has shown that amyloid proteinaceous materials contribute to support and conduct complex biological functions [6]. For all these reasons, amyloids, once exclusively affiliated as pathological and toxic structures, are now raising interest as biomimicry self-assembling class of biological elements for artificial materials production. Their potential engineering is a consequence of the study and comprehension of the supramolecular structuration [7].

**Figure 1:**
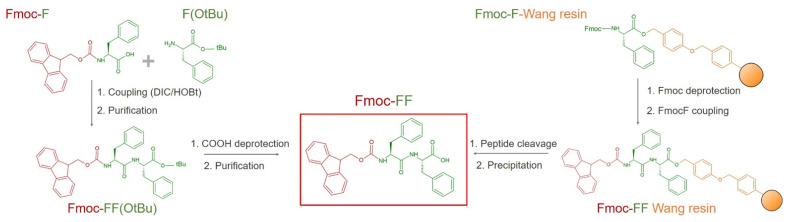
Scheme for the synthesis of Fmoc-FF (red rectangle) using liquid phase strategy (on the left) and solid phase peptide synthesis (SPPS) on the right. DIC: *N*,*N*′-Diisopropylcarbodiimide; HOBt: 1-Hydroxybenzotriazole.

Identified in the middle of the primary sequences of Aβ_1–40_ and Aβ_1–42_, diphenylalanine (FF) was recognized as the crucial aggregative motif in the Alzheimer’s β-amyloid polypeptides [8]. This simple homopeptide revealed the capability to self-organize efficiently into well-ordered tubular architectures with a long persistence length (~100 µm) because of π-π staking and H-bonding interaction networks [9]. Due to its chemical simplicity and versatility, FF rapidly became the paradigm for the study for peptide self-assembly. Moreover, modifying its very simple chemical structure, a plethora of FF analogues was proposed during in recent years [10,11,12,13,14,15,16,17,18]. Cationic FF differs from FF (or zwitterionic FF) for the amidation of the C-terminus. In the amide form, the C-terminus is unable to generate head-to-tail hydrogen-bound interactions and dipeptide preferentially self-assembles into nanowires [19]. With the rationale of generating a covalent attachment to fabricated gold electrodes for application in nano-devices, Gazit et al. designed the tripeptide Cys-FF in which the thiol group contributes to cross-linking phenomena and to the self-aggregation into nanotubes [20]. Other diphenylalanine analogues able to aggregate in micro-structures, such as flat plates, flattened micro-planks, and micro-rods, were obtained for progressive elongation of the Phe side chain with methylene groups (dihomophenylalanine (DiHpa); di-2-amino-5-phenylpentanoic acid (DiApp); di-2-amino-6-phenylhexanoic acid (DiAph)) [21]. With the aim to elucidate the supposed role of electrostatic interactions in the peptide backbone aggregation, in 2005 an FF derivative in which the N-terminal amine and the C-terminal carboxyl of the peptide are respectively acetylated and amidated (Ac-Phe-Phe-NH_2_) [20] were designed and synthetized. This uncharged peptide showed the capability to efficiently self-assemble into tubular structures. Consequently, the authors analysed a small library of analogues, Ac-Phe-Phe-OH, Boc-Phe-Phe-OH, Cbz-Phe-Phe-OH, and Fmoc-Phe-Phe-OH, in which only the amino group was blocked by using conventional solid phase peptide synthesis (SPPS) protecting groups [22]. The tert-Butyloxycarbonyl (Boc)-FF monomer analogue allows to obtain peptide spheres or small peptide particles when its HFIP stock solution is diluted in ethanol [23,24]. Instead, due to the additional stacking between the Benzyloxycarbonyl (Cbz) or 9-fluorenylmethoxycarbonyl (Fmoc) aromatic moieties, the two correspondent FF protected compounds self-assembled into fibrillary structures. Fibrillary structures of Fmoc-FF-OH showed an ultrastructure and dimensions extremely similar to the amyloid fibrils. Fmoc-FF dipeptide (see Figure 1 for the chemical structure) is one of the most studied ultra-short peptides for hydrogel (HG) preparation [25,26]. The main reason for this large interest is related to the possibility to obtain stable self-supporting hydrogels at pH values compatible with physiological applications, including tissue engineering and drug delivery. Additional applicative areas cover chemical catalysis, nanoreactors development, optical engineering, wound treatments, ophthalmic preparations, energy harvesting, antifouling, and biocompatible coating applications, optoelectronics, potential immuno-responsive agents, and absorbents systems for oil/water separation [27].

The chemical accessibility, biodegradability, biofunctionality and the possibility to adopt specific secondary, tertiary, or quaternary architectures represent additional advantages for this simple peptide building block, alone, in combination with different chemical entities or in different morphological shapes [28,29]. Moreover, it was observed that matrix structural and functional properties can be tuned opportunely by simply modifying the gelation kinetic and other experimental parameters (such as pH, temperature, and used solvent) [30,31]. This observation pushed the research towards the development of novel and alternative methods to achieve HG generation. On the other hand, novel peptide analogues, containing codified or non-codified amino acids, have been proposed as building blocks for the preparation of hydrogels with enhanced properties [32,33]. It is worth noting that although the identification of Fmoc-FF as a hydrogelator dates to 15 years ago, its behaviour is continuously studied. The aim of this review is to provide an overview of the different preparation methods reported until now for the fabrication of Fmoc-FF HGs. This study also notes how the used method can affect both the structural and mechanical properties of the obtained material.

## 2. Applicative and Biomedical Relevance of Fmoc-FF Hydrogel

In the plethora of peptide-based hydrogels, Fmoc-FF becomes a relevant system due to some specific advantages. With respect to other peptide-hydrogelators (e.g., RADA peptides, [34] MAX-1, [35], amphiphilic and no-natural containing sequences [36,37]), Fmoc-FF represents a simple and accessible chemical entity, commercially available from different companies. The structural simplicity of the molecules additionally allows its production in high purity and with contained costs, both related to a solid phase peptide synthesis (SPPS) [38] and to the synthesis in solution (Figure 1). In the SPPS, the product is obtained into a three-step synthetic route. Specifically, a preloaded Fmoc-F-Wang resin undergoes a Fmoc-F-OH coupling after a Fmoc-deprotection. The cleavage of peptide and its precipitation make available the final powder with an efficient scalability. Alternatively, the same molecule can be obtained in solution, taking advantage of the non-requested protection of the Phe side chain. In this case, a C-protected phenylalanine is coupled with a Fmoc-F-OH residue. Then, after the deprotection of the carboxylic acid, Fmoc-FF is precipitated and purified.

Another advantage of Fmoc-FF as a building block for hydrogel preparation is its fast kinetics of formation, which occurs in a few minutes. Moreover, the gel can be prepared by using both in vitro and in vivo friendly solvents (water, buffers, and cell media) [39]. Additionally, the optical transparency of the Fmoc-FF matrix is a benefit for a preliminary macroscopical evaluation in terms of homogeneity and correct formulation. Moreover, the mechanical properties and the tunability exhibited by the Fmoc-FF matrix make it compatible with extrusion, electrospinning, and filming deposition procedures [40,41]. Finally, the resulting hydrogel exhibits a good shelf stability. All these advantages make Fmoc-FF highly accessible for application in many research fields.

Early investigations carried out by Gazit and co-workers noted only the capability of the dipeptide to self-assemble into a rigid material with macroscopic characteristics of a self-supporting gel [26]. The hydrogel formation was achieved with a “solvent switch” methodology (*vide intra*), which consists into the dilution of an organic peptide stock solution (generally at 100 mg/mL) in water. In this specific case, HFIP (1,1,1,3,3,3-hexafluoro-2-propanol) was used as an organic solvent, diluted in water at a final concentration of 5 mg/mL (0.5 wt%) [26]. The pre-dissolution in HFIP is currently used as aggregative step for other aromatic peptide-based materials [42,43,44]. The so prepared materials were found syringable, shaped, and stable across a broad range of temperatures, over a wide pH range and in presence of aggressive chemical agents, such as guanidinium and urea. Due to the presence of hollow cavities in the supramolecular architecture, the possible use of Fmoc-FF as a reservoir was scrutinized by the encapsulation and release study of model drugs such as fluorescein (FITC) and insulin-FITC, indicating a retain of molecules of 5 kDa, and a slow release for small chemical entities [26]. The remarkable mechanical rigidity of this hydrogel compared with others physically cross-linked ones, the direct correlation between rheological properties and peptide concentration, and its capability to support Chinese Hamster Ovarian (CHO) cell adhesion led authors to suggest Fmoc-FF as a promising, tunable, and versatile scaffold for tissue engineering too. Simultaneously, the evidence reported that some Fmoc-protected dipeptides and amino acids are able to form scaffold materials [45,46,47], Ulijn and co-workers synthetized am Fmoc-based dipeptide library built as combination of glycine (Gly), leucine (Leu), phenylalanine (Phe), and alanine (Ala). Using a progressive decrease in the pH (from 8 to < 4, procedure that are reported as “pH switch” method), the authors noted that all the library members were able to form fibrous-based hydrogels. Only Fmoc-FF produced low-concentration HGs after lowering the pH to a physiologically relevant value [25]. Fmoc-FF gel was also tested for its ability to support proliferation and retention of phenotype bovine chondrocytes, both in 2D and 3D experiments.

Beyond tissue engineering and drug delivery applications, Fmoc-FF nanomaterials have been investigated as innovative materials in industrial and biotechnological fields. For example, in 2010 Rosenman et al. proposed self-assembled Fmoc-FF materials as biosensors for the detection of amyloid fibrils. From the analysis of the optical properties, it was proposed a shift from a 0D-quantum dot to a 2D-quantum well with a thickness around 1 nm. This observation demonstrates the capability of this self-assembled peptide to exhibit the same quantum physical phenomenon previously observed only for semiconductor crystals [48]. Moreover, to improve the biofunctionality and the biocompatibility of bio-based materials such as silica wafers, Fmoc-FF peptide was covalently anchored on its the surface. In this case, the AFM characterization pointed out the tendency of the aromatic dipeptide to self-assemble in nanorods with a mean radius ranging from 10 to 30 nm. The structural arrangement of the peptide on the silica surface allows a modification of the functional properties of the material in terms of angle of contact. As expected, the density of nanorods on the silica surface is strictly related to the concentration of the immobilized peptide [49]. Analogously to FF-based nanomaterials, dried Fmoc-FF peptide hydrogels revealed piezoelectricity under piezoresponse force microscopy. Due to their biomimicry nature, coupled to these electric properties, Fmoc-FF gels were also proposed as advantageous tools in application in which electrical stimuli are required (e.g., axonal regeneration). The non-centrosymmetric topology of the β-sheet rich fibres were quoted as the ascribable structural reason of the electric features of the material. Indeed, an overall polarization along the fibre axis is related to dipoles, running perpendicularly to the direction of the β-strands [50]. Recently, Fmoc-FF hydrogels and some of its cationic variants (Fmoc-FFKK, Fmoc-FFFKK, and Fmoc-FFOO, in which O is the one code symbol for ornithine) have been studied for the first time as antibacterial materials [51]. Gel preparation was achieved at a final peptide concentration of 2.0 wt%. Unsurprisingly, self-assembled cationic variants exhibited high ordered antiparallel β-sheet structures and low rigidity and viscosity. Fmoc-FF hydrogels showed significant antibiofilm activity preventing the growth of bacteria and biofilm of Gram-positive (*Staphylococcus aureus* and *epidermis*) and Gram-negative (*Pseudomonas aeruginosa* and *Escherichia Coli*) bacteria that can be localized on the medical devise surface.

Recently, Adler-Abramovich and co-workers reported the prominent capability of Fmoc-FF hydrogel to specifically encage oxygen molecules and restrict its movement in absence of metal ions [52]. Molecular dynamics computations highlighted that the binding of O_2_ is originated by specific interactions that take place between O_2_ and the interior surface of Fmoc-FF fibrils. The ability of the gel to encage oxygen suggests its potential application as a passive mean to maintain hydrogen production by the O_2_-hypersensitive enzyme [FeFe]-hydrogenase. These results leave envisage potential utilization of Fmoc-FF hydrogels in a wide range of O_2_-sensitive applications.

## 3. Structural Organization and Proposed Model

**Figure 2:**
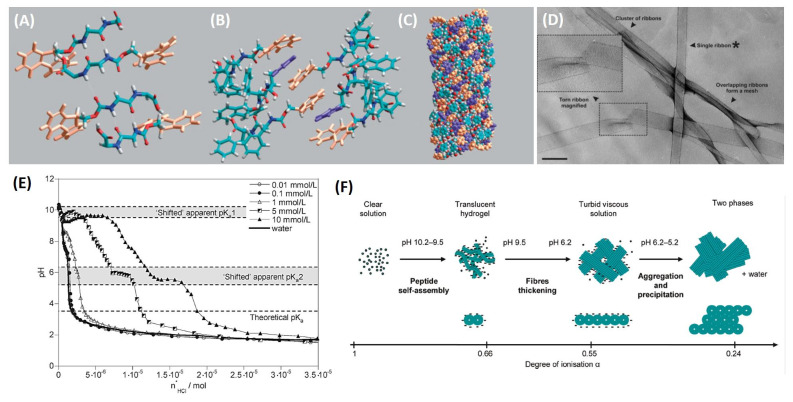
Structural model of Fmoc-FF peptides. (**A**) Dipeptide copies are arranged into β-sheet with an antiparallel orientation of β-strands. (**B**) π-stacked pairs due to the interlocking of fluorenyl groups from alternate β-sheets. (**C**) The final model obtained by energy minimization. In the model Fmoc and the phenyl groups are coloured in orange and in purple, respectively. (**D**) Transmission electron microscopy of Fmoc-FF xerogel (scale bar = 500 Å); the ribbon asterixed by authors was selected for other morphological analysis. (**E**) Titration curves of water and Fmoc-FF samples at different peptide concentrations (0.01, 0.1, 1, 5, and 10 mmol/L). (**F**) Mechanism proposed to explain the formation of Fmoc-FF aggregates as consequence of the pH decrease (figure adapted with permission for Refs. [53,54], Copyright 2009 American Chemical Society).

The growing interest around Fmoc-FF as innovative material also encouraged structural studies aimed to identify the model of aggregation into fibrillary networks. In 2008, Uljin and co-workers proposed for the first time the aggregation model for Fmoc-FF [53]. The model was designed on the basis of their experimental data collected by Circular Dicrohism (CD) and Fourier Transformed Infrared spectroscopies (FT-IR). Structural characterization highlighted an anti-parallel β-sheets arrangement of the peptide building blocks and anti-parallel π-stacking of the fluorenyl groups (Figure 2A,B). In more detail, in disassembled systems such as Fmoc-Phe, Fmoc group shows two dichroic prints at 307 nm and below 214 nm, without contribution in the far-UV region. On the contrary, in Fmoc-FF gels a negative pick centred at 218 nm is consistent with a β-sheet structure, implying that an ordered supramolecular structure is associated with the gel matrix. Gels caused a signal in the range of 304–308 nm, attributed to the π→π* transition in the fluorenyl-moiety too. Two other CD prints (a local maximum at 192 nm and a minimum at 202 nm) are indicative for α-helix transition associate to π→π* transition. The predominant β-arrangements were supported by FT-IR analysis too. Indeed, Fmoc-FF gels are characterized by two peaks in the amide I region (1630 and 1685 cm^−1^), compatible with an antiparallel organization of the peptides. All these structural requirements were well-satisfied by the model proposed by the authors: this model was based on a nanocylindrical structure (with an external diameter of ~3.0 nm, Figure 2C) formed by the interlocking through lateral π-π interactions of four twisted anti-parallel β-sheets. These fibrils, forming J-aggregates, were then shown to further self-assemble laterally, forming large flat ribbons under specific pH conditions, visible in TEM microscopy (Figure 2D). The scattering pattern of the Fmoc-FF-dried gel showed a series of diffractions compatible with the structural organization of the proposed model [53]. Based on the evidence that pK_a_ can significantly change as a consequence of protein and peptide self-organization phenomenon, Saiani et al. studied effect of pH on the Fmoc-FF self-assembly process [54]. Their results suggested that the self-assembly of Fmoc-FF building blocks is prompted by a lowering of the pH as consequence of two apparent pK_a_ shifts (of ≈6.4 and 2.2 pH units, respectively) above the theoretical pK_a_ value (3.5) (see Figure 2E). At high pH, where the Fmoc-FF building blocks are in their ionized form, the self-assembly is mainly forbidden. Then in correspondence of the first pK_a1_, at pH 10.2–9.5, both protonated and non-protonated molecules begin to self-assemble into paired fibrils (Figure 2F). The further pH lowering from 9.5 to 6.2 causes a decrease in the fibre surface charge that in turn brings to the formation of large rigid ribbons due to the lateral interactions of the fibres. Below pH 6.2 (between 6.2 and 5.2) the second apparent pK_a_ shift is observed, where a further aggregation of the ribbons occurs [54]. Recently, Yan et al. speculated on the possibility to achieve a conformational transition from a β-sheet structure to a helical one by a charge-induced strategy. To demonstrate their hypothesis, they studied the structural behaviour of Fmoc-FF hydrogel before and after the addition of Na_2_B_4_O_7_-EDTA buffer (pH = 8.5) [55]. Under these basic conditions, a structural transformation was detected from antiparallel β-sheet structures to a parallel helical one, imputable to the electrostatic repulsion between the negatively charged peptide molecules. This transition was further confirmed by computational simulations studies. The interaction energy analysis also highlighted the crucial role performed from the water molecule in stabilizing the Fmoc-FF^−^ helix nanofibril. Successively, the same authors also demonstrated that the addition of metal ions can induce structural transformation in Fmoc-FF from an amyloid-like β-sheet into a superhelix or random coil [56]. The peptide structural organization was found dependent from the types and ratios of metal ion/dipeptide through metal.

The inner size of the hydrogel cavity was estimated by Huppert et al. by a simple and non-destructive method based on the reversible photoproteolytic cycle of the photoacid 8-hydroxypyrene-1,3,6-trisulfonate (HPTS, pyranine) [57]. HPTS, a photoacid molecule with a triple negative nature, is able to transfer H^+^ ions with a time constant around 100 ps. The study was conducted on the experimental evidence that the parameter affecting the photocycles are related to water sphere radius in very small, confined volumes of media, as in hydrogel matrices. In the Fmoc-FF hydrogel the water cavity dimension between the fibril walls was found to be around 100 Å. This narrow size, inaccessible for living cells, suggests that in tissue engineering applications the scaffold peptide material is directly formed around the cells. Recently, the Point Accumulation for Imaging in Nanoscale Topography (PAINT) technique was used for the first time to 3D-image Fmoc-FF hydrogels in native conditions (no dry gel) and in absence of a direct labelling of the gel. PAINT images revealed the presence in the gel of fibres with a diameter of 50 nm and a mesh size ranged between 20 and 40 nm^2^ [58].

## 4. Preparation Methods

**Figure 3:**
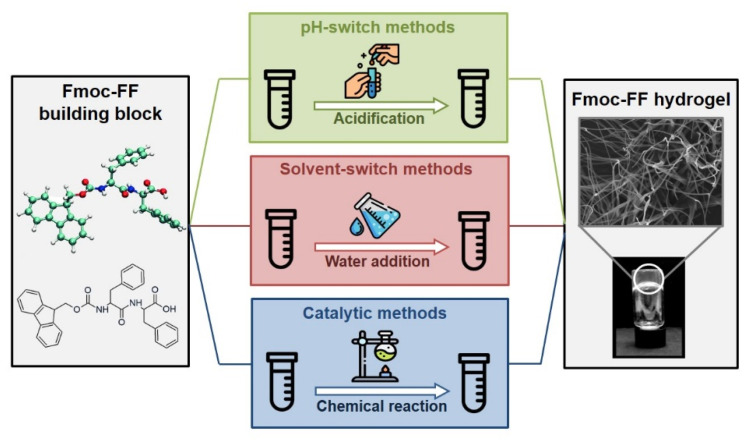
On the left, the molecular Fmoc-FF building block, reported both as 3D balls and sticks and as chemical formula. On the right, an inverted vial containing Fmoc-FF self-supporting gel and its TEM image. In the middle a schematic representation of three different methods (pH-switch, solvent switch, and catalytic one) commonly used for trigger gelation process.

Fifteen years of scientific research on Fmoc-FF have been pointed out that the local organization of this peptide fragment and its structural and macroscopic architecture is deeply affected by the preparation method and by the experimental conditions used to generate the supramolecular material. After all, it is well-known and intuitively predictable that the self-assembling environmental conditions and/or the self-assembling strategy can affect both the macroscopic and the microscopic structures of the resulting hydrogel and, in turn, its functional features [59,60,61]. Moreover, fabrication of uniform hydrogels is often hampered by the diffusion of peptide molecules and aggregates into water medium. As a consequence of this different hierarchical organization, the physicochemical characteristic (stiffness, matrix porosity, stability, opacity, and so on) and consequently the potential applications (tissue engineering, drug delivery, biocatalysis, and biosensors) of the material change. For example, mechanical properties of the hydrogel can change up to four orders of magnitude by modification of some parameter in the formulative process, such as temperature, peptide concentration, pH of the solution, and the addition of salt/additives [62]. In this contest it is relevant to understand the key factors that can allow obtaining the material with the desired properties. To describe and distinguish between the preparation methods available until now, in this paper they were classified into three classes (Figure 3), which are named as: (i) pH-switch method, (ii) solvent-switch method and (iii) catalytic method. All of them allow gel formation as a consequence of a change/modification of the initial conditions, introducing a trigger into the peptide solution. In the first two methods, nominally pH- and solvent-switch methods, the gelation often occurs rapidly (less than a second) in the neighbourhood where the mixing of the two different solutions takes place, and in certain cases they lead to the formation of an inhomogeneous hydrogel. However, originally proposed strategies have been opportunely modified to improve the homogeneity of the gel. Analogously, catalytic methods, in which the self-aggregation process can be kinetically and thermodynamically controlled, have been proposed. It is worth noting that the kinetic control on the self-assembly process can allow driving the aggregation towards a designed pathway and in turn, allow the formation of highly homogeneous hydrogels.

### 4.1. pH-Switch Method

The pH-switch method involves the dissolution of the peptide in an aqueous solution at elevated pH (around pH 10.5) followed by a solution pH lowering via HCl addition [53]. In this preparation method, the peptide solubilisation at high pH ensures the deprotonation of the C-terminal carboxylic acid. Then the progressive acidification allows its protonation until gelation.

Due to the potential cleavage of the Fmoc protecting group under basic conditions, a careful control of the pH value and of the permanence time of the sample in NaOH is required during the peptide dissolution. It is worth noting that a negligible percentage of the fluorenyl moiety (<1%) is lost after 10 min in this basic condition. It was observed that the acidification of Fmoc-peptide solution by inorganic acids such as HCl brings to the formation of an inhomogeneous hydrogel. This result was attributed to the kinetics of mixing being slower than the initial kinetics of gelation. Indeed, at a low pH value, fibril formation and gelation are often very fast (taking place in a time lower than a second), with the consequence that it is very difficult to achieve a uniform pH in solution before the gelation process starts. Reproducible and more homogeneous hydrogels were obtained using an optimized procedure, involving a progressive addition of acid followed by a heat/cool cycle [54]. The high temperature permits to dissolve the kinetically trapped aggregates that are formed during the acidification. As described, the assembly of Fmoc-FF building blocks occurs with the appearance of two apparent pK_a_: the first at pH 10.2–9.5, and the second at pH 6.2–5.2. It should be emphasised that hydrogels free from undissolved peptides exhibit a G’ modulus of 1–10 Pa, which is significantly lower than G’ (10^4^ Pa) of hydrogels undergoing the heating step.

**Figure 4:**
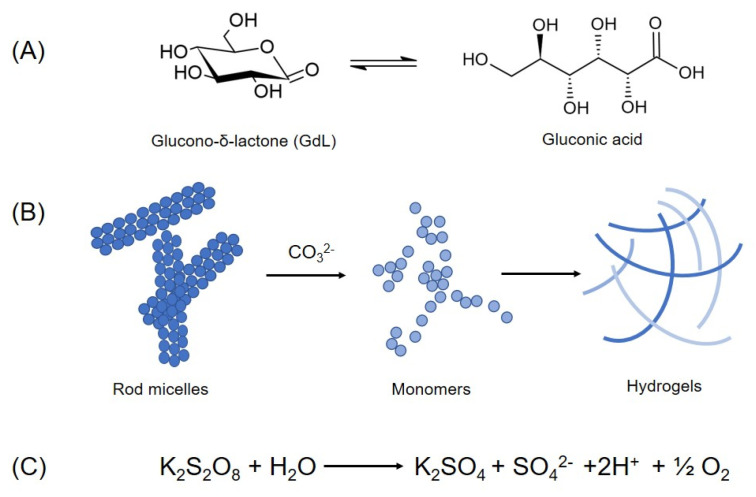
Preparation of Fmoc-FF hydrogel using three variants of the classic pH-switch method employing HCl: (**A**) decrease in pH induced by the slow hydrolysis (≈18 h) of the glucono-δ-lactone (GdL) to gluconic acid; (**B**) the colloid method in which the hydrogel is obtained by a progressive colloid-to-hydrogel transition triggered by adding of Na_2_CO_3_ to the colloid solution; (**C**) decrease in pH allowed by the decomposition of the K_2_S_2_O_8_ in oxygen gas and protons.

It is also important to pay the attention to each step during the gel preparation. Indeed, it was demonstrated that the method utilized to agitate the sample during and after the gelling transition can dramatically affect both the nano-scale morphology and the mechanical properties of the gel. The effect of the agitation (low shear and high shear) was evaluated on different pure and mixed hydrogels (100% Fmoc-FF, 70/30 Fmoc-FF/Fmoc-GG, and 50/50 Fmoc-FF/Fmoc-GG), all of them prepared according to the pH-dependent procedure [30]. In this study, low shear gels exhibited a higher storage modulus (≈4000 Pa) with respect to the high shear ones (≈1000 Pa). The different mechanical properties have been related to the morphology of gels obtained in the two shear modalities: high shear samples are more prone to lateral aggregation, whereas low shear ones to a regular form of entanglement. Successively, Adams and co-workers introduced a novel strategy in which the decrease in the pH is prompted by the slow hydrolysis of the highly soluble glucono-δ-lactone (GdL) to gluconic acid (see Figure 4A) [63]. The long timescale for the hydrolysis (≈18 h) allows a slow and homogeneous pH change, which minimizes the solvent mixing effects with the consequential obtainment of homogeneous and reproducible gel. According to this procedure, only a single apparent pK_a_ of 8.9 was detected. The final pH of the solution can be modulated according to the amount of GdL added to the peptide. The study demonstrated that the addition of two equivalents of GdL with respect to the peptide allows reaching a final pH of 3.6–3.9, which is very similar to the pH reached by adding HCl. However, the pH begins to equilibrate after ≈350 min and is fully equilibrated only after 24 h. The combination of the GdL-mediated pH trigger method with the cryogelation at a sub-zero temperature (−12 °C) allows the formation of macroporous Fmoc-FF hydrogels [64]. In the conditions here described, the water crystallization occurs and the Fmoc-FF molecules, concentrated in the remaining non-frozen liquid phase, gelate with a structure having a pore size in the range of 10–100 µm. Even if the pore walls of cryogels are characterized by a close packing of the fibres, they exhibit low mechanical stability with respect to classical hydrogels. This low stability was attributed to the heterogeneous structure in the cryogel. To further improve the homogeneity and the reproducibility of the hydrogel, in 2013 Ding et al. described two novel pH-triggered procedures for achieving Fmoc-FF hydrogel fabrication [65]. In the first approach, termed “the colloid method”, hydrogels were obtained by a colloid-to-hydrogel transition process (Figure 4B). Initially, the amphiphilic Fmoc-FF dipeptide spontaneously self-assembled in water solution in stable, rod-like micelles with a radius and a length of ≈15 and ≈180 nm, respectively. Successively, the slow formation of hydrogel (≈20 min) was triggered by adding one equivalent of a weak base such as Na_2_CO_3_ to the colloid solution. The addition of the base causes the progressive deprotonation of the carboxylic functions of the dipeptides present at the surface layer of the micelles. In this context, micelles serve as a trap to regulate the amount of Fmoc-FF released in the solution and hydrogels can be prepared also at pH > 9. Instead, the second approach was based on the decomposition of the K_2_S_2_O_8_ in oxygen gas and protons triggered pH decrease (reaction is reported in Figure 4C). This method exhibits several advantages compared with other methods used to lower pH such as the hydrolysis of GdL. It permits to control the amount of protons released and hence the final pH of the solution. Moreover, due to the dependence of the potassium persulfate degradation from the temperature, both the gelation kinetic (from 5 min at 90 °C to 4–5 days at 25 °C) and the structural properties of the hydrogel can be programmed, as well.

### 4.2. Solvent-Switch Method

**Figure 5:**
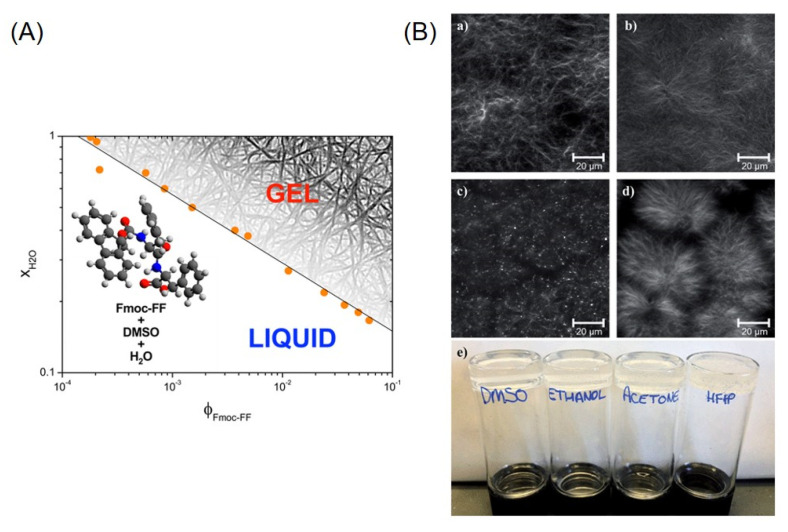
(**A**) Phase diagram molecular gels produced by Fmoc-FF using DMSO/H_2_O solvent-switch method (figure adapted with permission from Ref. [67], Copyright 2014 American Chemical Society). (**B**) Fmoc-FF matrices stained with Nile Blue in confocal microscopy analysis (*ϕ*solvent = 0.3) using the solvents (**a**) DMSO (**b**) ethanol (**c**) acetone (**d**) HFIP, (**e**) macroscopical gel picture after 24 h from their preparation (at same *ϕ*solvent without staining) (figure reproduced from Ref. [71] with permission from the Royal Society of Chemistry).

Beyond the pH-switch method, Fmoc-FF hydrogels can be also prepared with the solvent-switch method. This is based on the dissolution of the peptide in an organic solvent at high concentration. Then, gelation is trigged by adding water to the solution, so creating a three-component (peptide/solvent/water) system. Initially, Gazit and co-workers used HFIP as a solvent to dissolve Fmoc-FF [26]; later, other organic solvents able to solubilize Fmoc-FF peptides were used as an alternative to HFIP. Specifically, the first solvent used in place of the HFIP was dimethyl sulfoxide (DMSO). TEM images collected on Fmoc-FF gels prepared in DMSO/water confirm the presence of small-entangled fibres with a diameter of ≈10 nm, smaller than the wavelength of visible light. Moreover, the storage modulus value (10^4^ Pa) found for these gels was similar to that one for HFIP/water preparation [66]. In 2014, Dudukovic and Zukosky established a range of concentrations under which a Fmoc-FF solution in DMSO can form hydrogels upon mixing with the water and studied the mechanical properties of the gels prepared in the different conditions [67,68]. Their results allowed to delineate a well-defined line of gel transition in a plot of water concentration as a function of *ϕ* (namely the particle volume fraction) and, pointed out that rigid gels can be obtained at a low Fmoc-FF volume fraction (*ϕ* < 1%) and under addition of a small amount of water to DMSO (Figure 5A). The capability of the peptide to gel also under addition of reduced quantity of water was recently demonstrated to be due to the existence of disordered oligomers and profibrils into the DMSO solution [69]. Moreover, a deep investigation on the Fmoc-FF gelation kinetics revealed that upon addition of water to DMSO solution of Fmoc-FF, initially the dipeptide self-assembles into a metastable non equilibrium state composed of spherical clusters of diameters of 2 µm, followed by a rapid rearrangement (below 5 min) into a fibrous network. The aging of the sample (up to 4 h) allows a further evolution of the gel towards a steady state in which there is the formation of a highly uniform network composed of thin fibres with a mean diameter between 5 and 10 nm. These dimensions are smaller than the wavelength of the visible light and are the reason why the gel appears transparent. The high uniformity of the fibrillary network causes an increase in the gel rigidity and provides long-term stability (years) to the gel. These studies also demonstrated the mechanical and thermal reversibility of the gels over time. Successively, the same authors speculated that the fibres in the gel can be treated as an equilibrium crystalline state, in which the gelation process is a first order phase transition resulting in the nucleation and growth of elongated anisotropic crystals. It was observed that an increase in water with respect to DMSO brought an increase in the strength of attraction between the peptide molecules that in turn can be translated into a fast nucleation rate and quasi-one-dimensional crystal growth [70]. This transition from spherulitic structures to a fibrous network was also observed by Adams and co-workers for Fmoc-FF hydrogels prepared using other polar protic or aprotic solvents (Figure 5B) such as ethanol, acetone, and HFIP in place of DMSO [71]. However, it seems that the choice of the solvent can affect the morphology of the final network and in turn rheological properties and mechanosensitivity of the final hydrogel. These differences are originated by the control exercised by the solvent on the morphology of the fibre network. For example, at a *ϕ*_solvent_ of 0.3, gels prepared in ethanol exhibit a more uniform network with respect to gels prepared in DMSO or HFIP. This high uniformity is associated with a high rigidity and a poor capability of the hydrogel to recover their mechanical strength from shear. It is worth noting that the transition from opaque to limpid state into the peptide solution is never observed for samples prepared by the pH-switch. This evidence clearly indicates that there are significant differences in the self-assembling process occurring for the two procedures.

Later, Dudukovic et al. also explored the phase behaviour of Fmoc-FF in other solvent systems (DMSO/H_2_O, MeOH/H_2_O, and toluene). According to the Adam’s results, the authors demonstrated that gel formation can be induced also in apolar solvents and that the Fmoc-FF phase behaviour is directly correlated to the balance between the inter- and intra-molecular interactions. Indeed, it was observed that different solvents allow the formation of fibres with a different molecular order and that Fmoc-FF exhibits polymorphism in some solvents where metastable anisotropic crystals evolve towards crystal aggregation with no preferential axis of growth. Different fibre populations can co-exist within one system and the switch between these states depends on the stability of each conformational state and on the height of barrier between the two free energy minima (Figure 5) [72]. As an alternative, hydrogels can be prepared by using buffered solutions at physiological pH. However, also the ratio of DMSO to H_2_O (*ϕ*_DMSO_) and the choice of the buffers used in selected systems can affect the rheological properties of the hydrogel [65].

### 4.3. Catalytic Methods

The catalytic process is an interesting methodology that allows directing the self-aggregation process towards structurally diverse self-assembled materials, inaccessible via classical self-assembly. This approach consists of converting precursors unable to self-assemble into building blocks able to do. Usually, this conversion can be achieved by enzymatic removal or hydrolysis of a charged or steric groups that avoids the aggregation. In this case, the nucleation site and the early stage grown mechanism are spatially confined at the site of catalytic centre. Two examples of self-assembly for Fmoc-FF and for several its analogues (FY, YL, VL and FL) were reported by the Ulijn’s group [73,74]. Initially, they demonstrated the use of thermolysin, a non-specific endoprotease to catalyse in a reversible way the peptide bond formation between Fmoc-F and several dipeptides (G_2_, F_2_, L_2_) or amino acid esters (L-OMe, F-OMe) [73]. After the bond formation, the peptide self-assembles in a spatiotemporally controlled manner, with the enzyme favouring the spatial confinement of structure grown during the early stage of the self-assembly process. On the same set of peptides, they also reported the self-assembly catalysed by subtilin, a hydrolytic enzyme from *Bacillus licheniformis*. It was observed that the enzyme concentration (ranged between 1.5 and 36 units) strongly affects the self-assembly kinetics and in turn, the supramolecular order degree, and the functional properties of the final material [74]. Chemical catalysis, achieved by starting from phenylalanine and its Fmoc-derivative and EDC/NHSS as catalysts, was described as an alternative approach with respect to the biocatalytic ones [75]. In this procedure, Fmoc-F was activated in DMSO using EDC/NHSS, then Fmoc-FF was quickly obtained by simply adding the intermediates into an aqueous solution containing phenylalanine. The continuous generation of Fmoc-FF due to the chemical reaction and its spontaneous aggregation into entangled nanofibers allows the formation of a self-supporting and a homogeneous hydrogel. At the end of the gelling process, the catalysts were removed by washing the hydrogel with deionized water. Later, several Fmoc-FFF tripeptide, containing phenylalanine residues in *L* or *D* configuration, were synthetized by lipase-catalysed reversed hydrolysis reaction between a Fmoc-amino acid and a dipeptide [76].

## 5. Conclusions

Most of the peptide hydrogelators described in literature are able to gelate in only one well-defined condition, which is related to their primary sequence in terms of sterical hindrance, hydrophobicity, and polarity of amino acids. On the contrary, Fmoc-FF-based hydrogels can be prepared using different conditions of solvent, pH, temperature, ionic strength, and shear. From a predictive point of view, the versatility of Fmoc-FF was not expected on the basis of its very simple chemical nature. In this contest, many attempts have been made to understand the molecular mechanisms enabling the Fmoc-FF self-aggregation. The possibility to change preparation conditions and experimental variables allows to modulate in a controlled manner the structural properties and morphology of the resulting material. This versatility envisages a relevant number of applications in a variety of fields for this ultra-short peptide.

## Data Availability

Not applicable.
